# Comparative analysis of pain management protocols in pediatric and adult emergency medicine: a narrative review

**DOI:** 10.3389/fpubh.2026.1795766

**Published:** 2026-04-21

**Authors:** Ismail Abdullah, Sariya Khan, Syeda Hashim, Fatimah Shakeel, Mariam Amro, Dalaa Sheikh Saleh, Fatma E. Hassan

**Affiliations:** 1 College of Medicine, Alfaisal University, Riyadh, Saudi Arabia; 2 General Medicine Practice Program, Batterjee Medical College, Jeddah, Saudi Arabia; 3 Department of Physiology, General Medicine Practice Program, Batterjee Medical College, Jeddah, Saudi Arabia; 4 Medical Physiology Department, Kasr Alainy, Faculty of Medicine, Cairo University, Giza, Egypt

**Keywords:** emergency medicine, multimodal analgesia, pain assessment, pain management, pediatric vs. adult

## Abstract

**Background:**

Pain management in emergency medicine forms a critical aspect of the care provided to the patients and there are significant differences in the protocols tailored to pediatric and adult populations.

**Aim of the study:**

This narrative review aimed to compare and assess pain management strategies in pediatric versus adult emergency care, considering physiological differences and clinical approaches. The review synthesized evidence from studies conducted across global emergency department settings to provide broadly applicable insights.

**Materials and Methods:**

This literature review included many published studies till December 2025. The databases used include Google Scholar, PubMed and BMC Library using the following keywords “*Pain Management, Pain Assessment Tools, Multimodal Analgesia, Pain Pathways, Emergency Medicine*”.

**Results:**

There has been a clear point of divergence between pediatric and adult patients with respect to pain treatment as far as the emergency department is concerned. Pediatric patients rely on age-independent scales for evaluation, weight-adjusted drug administration methods, balanced opioid use, and a pharmacologic and nonpharmacologic approach that is liberal with regards to distraction therapy, topical analgesics, and regional analgesia. Adult treatment involves standardized scales for evaluation and a liberal pharmacologic approach for analgesics, opioids, adjunct analgesics, and multimodal analgesics.

**Conclusion:**

Pain management outcomes go beyond immediate comfort to impacts on psychological well-being, quality of life, and even recovery trajectory, thus supporting comprehensive and patient-centered care.

## 1 Introduction

While pain remains one of the most frequent presenting chief complaints among patients going to emergency departments, it still poses a very daunting task in management (1). In general, an improvement in pain improves the outcome, reduces anxiety and prevents acute pains from transitioning to chronic. The management of pain, however, remains very variable within the emergency settings because of a huge amount of variation regarding the differences among patient demographics, their underlying medical conditions, and physiologic responses (2). Special challenges in the management of pain are created by both pediatric and adult populations in the emergency department. In pediatric patients, consideration needs to take into account developmental stages, communication barriers, and pharmacokinetic-pharmacodynamic differences. Adults span a wide variation in conditions, usually complicated by comorbid states and polypharmacy, requiring individualized pain management. These variations make it very important to have evidence-based protocols that will help in providing the best care and minimize risks for both groups (3). Understanding the differences in pediatric versus adult pain management protocols is imperative in the delivery of appropriate, safe, and effective care according to the age of the patient.

Therefore, this study aimed to analyze and compare pediatric and adult pain management protocols in emergency medicine, identifying key similarities, differences, and areas for improvement. This review synthesized evidence from global emergency department settings to provide insights that can inform both high-resource and resource-limited healthcare systems. The findings aimed to support the development of more standardized, yet adaptable pain management approaches tailored to diverse patient populations.

## 2 Materials and methods

This narrative literature review explored published evidence on pain management strategies in pediatric and adult emergency medicine. The review focused on studies conducted in emergency department settings globally, without restriction to a specific geographic region.

A literature search was performed using PubMed, Google Scholar, and the BMC Library, including studies published from database inception until December 2025. Keywords used in the search included “pain management,” “pain assessment tools,” “multimodal analgesia,” “pain pathways,” and “emergency medicine.”

Studies were included if they addressed pain assessment or pain management strategies in pediatric (0–18 years) or adult (>18 years) populations within emergency medicine settings. Studies involving both male and female patients were eligible. Articles were excluded if they were not published in English, were conducted outside emergency care contexts, or focused on highly specialized populations not directly relevant to emergency medicine (e.g., oncology-related pain, palliative care, or postoperative pain managed exclusively in surgical settings).

Data extraction included study aims, population characteristics, pain assessment tools, management strategies, and key outcomes. The results of the included studies were narratively synthesized and did not contain a thorough tabulated dataset or a methodical listing of all included studies. Consequently, rather than using quantitative aggregation, the synthesis was focused on thematic analysis of the literature. Tables and textual descriptions were used to present key findings and identify similarities and differences between pediatric and adult pain management practices.

## 3 Physiological differences in pain perception between pediatrics and adults

### 3.1 Pain pathways and development

Pain pathways, which are intricate and dependent on pain etiology, duration, and site, have been extensively studied and outlined in literature (4–7). A basic overview of pain pathways starts at nociceptors, from which the primary afferent (first order) neurons that sense and transmit pain sensations arise. Nociceptors found in the dorsal root ganglion and trigeminal ganglia receive impulses from the body and face, respectively, that then travel along either Aδ or C fibers. Aδ fibers are myelinated and conduct pain of mechanical or thermal origin at high speeds. They are associated with fast pain of sharp burning, pricking, or stabbing nature. On the other hand, C fibers are unmyelinated and conduct slow response burning or dull, aching pain (of various origins) at relatively slower speeds. These receptors are generally activated either directly through irritation or indirectly through inflammatory mediators as hydrogen ions, sodium ions, serotonin, cytokines, bradykinin, histamine, prostaglandins, and leukotrienes released secondary to surrounding tissue damage (7, 8).

After an action potential is conducted through the first order neuron, it is transmitted to a second order neuron in the dorsal horn (DH) of the spinal cord that ascends in one of three tracts: neospinothalamic, paleospinothalamic, or archispinothalamic (9). The neospinothalamic tract carries sensations of acute, localized pain. In the case that this pain arises from the body, second order neurons from the DH cross contralaterally and ascend via the lateral spinothalamic tract to terminate in the ventral posterolateral (VPL) and ventral posterior inferior nuclei (VPI) of the thalamus. From there, the third order neuron transmits pain sensation to the somatosensory cortex for additional processing and pain perception. Facial and intraoral pain begin with first order neurons located in the trigeminal ganglion that then enter the pons and descend to the medulla to synapse with the spinal trigeminal nucleus. After crossing, second order neurons of different fiber types (Aδ or C) terminate in different areas of the thalamus, whose third order neurons relay in the somatosensory cortex (4, 9, 10). The paleospinothalamic tract, by contrast, results in the sensation of poorly localized, chronic pain. Second order neurons of this tract project bilaterally to terminate in the reticular formation, periaqueductal gray area, tectum, and intralaminar (IL; and it is the parafascicular-centromedian complex) nuclei. The IL then relay in the somatosensory cortex, brainstem nuclei, and limbic areas, which contribute to the emotional and visceral responses associated with pain. Lastly, the archispinothalamic tract also plays a role in emotional and visceral responses to pain, as it projects bilaterally to ascend and descend within the spinal cord to reach the IL nuclei, with collaterals to the hypothalamus and limbic system (9).

**Figure 1:**
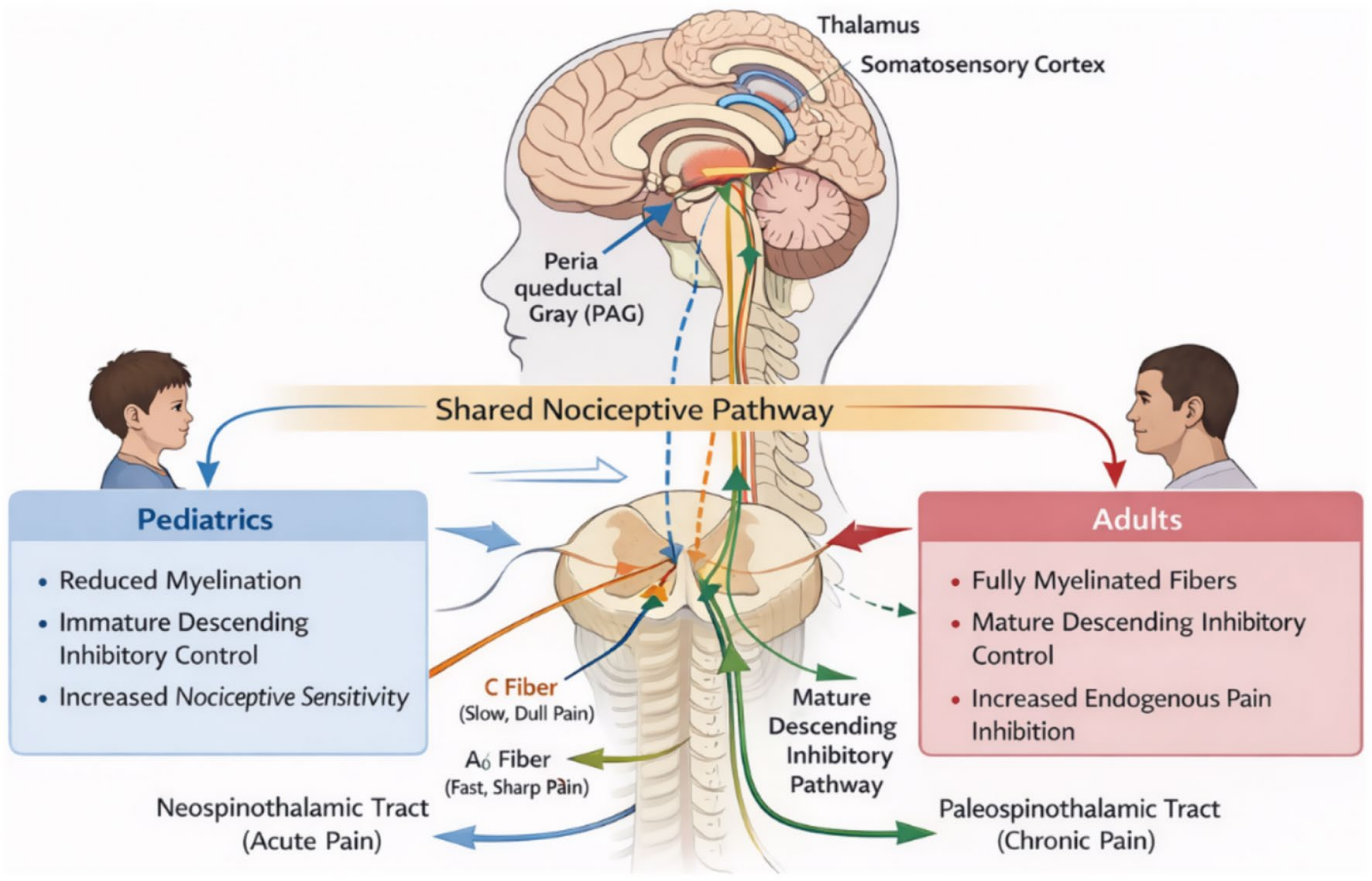
Comparison of pain pathways in pediatric and adult populations. [Created by the authors using generative artificial intelligence (AI) based on published literature on nociceptive pathways].

There has not been any significant variation in the essential anatomic architecture of pain pathway in humans with age, although some developmental variations have been documented in pediatric and adult populations. There has not been any variation in the physiology of children or adults in the peripheral or central pain pathway, nor in pain activation in both children or adults, or in any of the mechanisms of pain in any individual character wise, i.e., “nociceptor activation in both children or adults, or in any of the mechanisms of pain in any individual character wise.” However, these pathways play different roles based upon the differences associated with maturation. For pediatric populations, for instance, no myelination between nociceptive fibers as well as higher densities of more developing inhibitory descending pathways play a more substantial role in sensitivity to pain when compared to adults (4, 11, 12) (Figure 1). For pediatric populations specifically, unlike adults, immature descending pathways inhibitory to pain, excluding those from supraspinal structures such as those affecting the periaqueductal gray matter and rostroventral medulla, may heighten sensitivity to pain by reducing endogenous inhibition (12).

### 3.2 Variability in pain thresholds

Pain thresholds are critical components of pain perception; they are defined as the lowest intensity at which a stimulus is regarded as painful. This threshold is rather stable across individuals for certain stimuli; for example, most people perceive pain when a heat stimulus is approximately 50 °C. Similarly, unless in disease situations, mechanical pressure usually causes pain at equal amounts among individuals (13). These disparities cannot be attributable only to biased reporting or variances in pathology, since pain sensitivity may be reliably assessed using experimental pain stimuli. Sensitivity to various experimental pain modalities is often poorly correlated, indicating that different genetic and environmental factors likely influence individual pain responses (14). Pain sensitivity varies depending on certain gene polymorphisms. Variations in genes encoding opioid receptors and inflammatory mediators, for example, may have a major influence on an individual’s pain threshold and susceptibility to chronic pain (15). Single nucleotide polymorphisms (SNPs), which are combinations of inherited SNP alleles, may either enhance or reduce pain sensitivity (16). Another widely investigated potential gene is catechol-O-methyltransferase (COMT), which is known for inactivating neurotransmitters such as dopamine and norepinephrine and has been connected to differences in experimental and clinical pain perception (17).

Pain perception is also influenced by age differences. According to research, pain sensitivity diminishes as children grow into adults, with one study seeing a fast rise in cutaneous pain thresholds up to about the age of 25 (11, 18). This enhanced sensitivity throughout development may be due to processes in both the peripheral and central nervous systems. For example, adolescents may demonstrate a higher density of intraepidermal nerve fibers compared with adults, which can increase nociceptive input to the central nervous system. However, as individuals mature, the progressive development of descending inhibitory pathways and improved cognitive modulation of pain contribute to a gradual increase in pain threshold with age (12, 18, 19). Furthermore, adolescence is a pivotal era for brain development, with major morphological and functional changes in regions such as the amygdala, which influences emotional sensitivity to pain. Furthermore, areas such as the prefrontal cortex and posterior parietal cortex, which are critical for pain regulation, continuously maturing throughout this period (12).

The way the brain processes nociceptive information has a substantial impact on pain perception. Functional imaging studies show that several brain areas, such as the anterior cingulate cortex and insula, are engaged in the emotional and cognitive aspects of pain (18, 20). Variations in activity in these areas may result in difference in pain perception and reaction. Furthermore, hormonal factors, especially those secreted during stressful conditions, affect pain perception (20). High cortisol levels, for example, might enhance pain sensitivity, as seen in premenstrual syndrome and chronic pain syndromes. Consequently, a prolonged stress reaction may be linked to cortisol dysregulation and increased pain perception (21).

Also, inflammatory processes may dramatically amplify pain perception. Pro-inflammatory cytokines secreted after injury or during illness tend to lower pain thresholds, exacerbating the overall pain sensation. Individuals with chronic inflammatory disorders often have increased pain sensitivity as a result of ongoing stimulation of nociceptive pathways (20, 21). During inflammation, responses to hazardous stimuli may be heightened, known as hyperalgesia, or even minimal normally non-painful stimuli might cause pain, known as allodynia (22). Unresolved inflammation may contribute to persistent pain that lasts long after the primary injury has healed. This increased sensitivity associated with inflammatory pain is partially due to the nervous system’s plasticity, which serves as an adaptive response for continuous nociceptive impulses (23).

Finally, psychological and environmental variables contribute significantly to pain perception (24). Anxiety, anger, and sadness are more common in patients with negative moods, and they connect with higher degrees of clinical pain. Negative mood states predict not just the intensity of acute pain, but also greater pain sensitivity in laboratory conditions. This interaction between psychological variables and pain emphasizes the complexities of pain perception and the need for a comprehensive knowledge of its diversity (25).

## 4 Pain assessment tools

### 4.1 Pediatric pain scales

Pain assessment in pediatric patients cannot always rely on the same self-reporting methods used in adults, particularly in non-verbal infants and younger children, due to limited communication abilities. A number of pediatric pain assessment tools have been developed and tested by researchers and clinicians. Common examples include the Face, Legs, Activity, Cry, Consolability (FLACC) Scale, the Wong-Baker FACES Scale, and the Numerical Rating Scale for older children (26).

The most commonly used pain assessment tool is the FLACC Scale for patients who cannot speak due to their age. There is a five-category behavioral observation with scores ranging from 0 to 2. The cumulative score ranges from 0 to 10 which shows no pain at a score of 0 and severe pain at 10. This is especially helpful in a postoperative setting where non- verbal signs are important to identify pain (26, 27). The most commonly used pain tool for children above the age of 3 is the Wong-Baker FACES Pain Rating Scale. This scale offers a series of faces that range from smiling-no pain-to crying-severe pain. The child chooses the face that best describes their pain. This is an easy tool to be utilized for a child, but it does have limitations, especially with younger children who may point to faces based on emotions and not due to physical pain (28).

For older children, usually over the age of 8, the Numeric Pain Rating Scale (NPRS) is a simple tool where the children are required to grade their pain on a scale ranging from 0, for no pain, to 10 as the worst. It is simple but depends on the child’s notion and meaning of numerical values, which even then could pose a challenge with some younger or developmentally delayed children (29). Some of the challenges associated with pediatric pain assessment involve non-verbal communication and the stage of development.

Non-verbal infants and toddlers exhibit pain through facial expressions, crying, and movement of the body, therefore making observation tools such as the FLACC Scale useful for caregivers and healthcare professionals. As children mature and their cognition improves, the Wong-Baker FACES and NPRS become valuable tools (28, 29).

### 4.2 Adult pain scales

Assessment tools often rely on self-report in adults because they have the ability to describe their pain in great detail. Some of the common pain scales used on adults include the Visual Analog Scale (VAS), NPRS, and McGill Pain Questionnaire. The VAS is a simple yet powerful tool. Patients are asked to put a mark on a 10 cm line representing their pain, with one extremity of this line labeled “no pain” and the other “worst possible pain”. The distance, in centimeters from the “no pain” extreme to the marked point, is measured, hence giving a score out of 10. This tool is efficient for measuring the intensity of pains but can be quite problematic for patients suffering from cognitive or motor impairments (30).

The NPRS requests that the patients rate their pain, similarly to its pediatric counterpart, on a continuous scale ranging from 0 to 10. Because of this simplicity, it sees very frequent clinical use, though it is generally less effective in patients with cognitive decline or difficulty communicating (31). McGill Pain Questionnaire (MPQ) is an elaborated tool for assessing the intensity and quality of pain. With the use of a list of descriptors, such as throbbing, stabbing, and burning, it captures the sensory, affective, and evaluative dimensions of pain. MPQ provides a more complete understanding of the pain experience of the patient but may be time-consuming and burdensome to administer and complex to complete, especially in cases of limited literacy or cognitive impairments, for some patients (32). In these cases, the assessment can be performed with observational tools, such as the Pain Assessment in Advanced Dementia (PAINAD) scale, which is based on overt behaviors, like facial expressions and vocalizations (33).

## 5 Pain management protocols

### 5.1 Analgesic options

Pain protocol options are of utmost importance and have to be tailor-made according to the need, characteristics, and condition of each patient. The pharmacologic intervention for pediatric patients consists mainly of over-the-counter drugs that include acetaminophen and ibuprofen because they are widely used to treat mild and moderate pains due to their high safety profile and ease of formulation in liquid preparations (34). These are usually the first line in minor injuries or post-immunization pains. In serious or moderate pains, the use of opioids like morphine or fentanyl involves treatment but is done with many precautions owing to increased susceptibility in children to side effects, majorly being respiratory depression (35). In pediatric practice, regional anesthesia has gained more favor as a modality offering excellent localized pain relief with reduced systemic opioid exposure, such as nerve blocks with agents like bupivacaine. Of equal importance in pediatric care are the non-pharmacological methods: the application of topical anesthetics like eutectic mixture of local anesthetics (EMLA) cream before needle procedures, or the use of child-friendly gadgets like Buzzy to distract from painful stimuli. The range goes up owing to increased tolerance potential of the adult human body towards many varieties of pharmacological agents (36).

For adults, nonsteroidal anti-inflammatory drugs (NSAIDs), such as ketorolac, are the standard due to its strong anti-inflammatory action; thus, suited best in conditions such as musculoskeletal injuries or renal colic. Acetaminophen is usually employed in mild pain as a single agent, while with opioids, it is in heavy doses to achieve a synergistic effect. In acute severe pain, particularly with multiple injuries with fractures and burns or with severe postoperative conditions, opioids remain the cornerstone in the management but must always be given with strict caution because of the abuse, addictive properties, or adverse events, like nausea and sedation (37). Adjunctive therapies extend the pharmaceutical options: gabapentinoids for neuropathic pain, corticosteroids for inflammatory conditions, and antidepressants for chronic pain syndromes.

Multimodal analgesia has become widely adopted in both pediatric and adult populations, involving the combined use of analgesic agents from different pharmacological classes that target multiple pain pathways simultaneously to enhance pain control (38). For example, the combination of acetaminophen, NSAIDs, and local anesthetics may confer improvement in analgesia by decreasing opioid requirements. In the pediatric population, these multimodal approaches may combine pharmacologic treatments with non-drug strategies, such as distraction or physical comfort measures, while in adults they may be complemented by therapies such as acupuncture or physical therapy. Emergency protocols continue to change along with the shifting research, new pharmacological developments, and evolving concern over the opioid-related damage-an interplay of efficacy versus safety in every patient. It goes to show how analgesic options, their choice, and application could better meet the need for personalized care in pain management with respect to particular demographic features, while providing the best and humane care to pediatric and adult patients alike in the emergency setting (38, 39).

### 5.2 Non-pharmacological interventions

As the potential long-term complications, addiction risks, and side effects of opioid use have become more widely recognized, there has been a significant shift toward the investigation of non-pharmacological interventions as adjuncts or alternatives to conventional pharmacological treatments, such as opioids and NSAIDs. Nonpharmacological approaches augment, rather than replace, pharmacological therapies. These strategies are highly effective for treating mild to moderate pain (40). They may be used alone to treat low-intensity pain or in combination with pharmacological treatments to treat moderate- to high-level pain (41).

Cognitive Behavioral Therapy (CBT) is used to alleviate chronic pain and is becoming more popular in acute care. CBT stresses on emotional responses to pain as well as coping techniques via the modification of pain-related beliefs and behaviors (42). This technique is particularly successful in treating pain-related anxiety, a common co-occurring symptom among emergency patients (43). Emergency departments (EDs) are incorporating mindfulness and relaxation techniques, including guided imagery, progressive muscle relaxation, and deep breathing exercises, into their pain management protocols. These strategies aim to shift patients’ attention away from their suffering while also encouraging self-awareness and relaxation (42, 44). Studies have indicated that mindfulness-based therapies may reduce anxiety and pain perception, especially in the ED. According to research done by Wipplinger et al. (2023), individuals suffering acute pain saw a decrease in opiate intake, anxiety, and pain severity as a consequence of these strategies. When mindfulness activities are delivered by qualified personnel, patients are often able to better control their pain, without the need for rapid pharmaceutical interventions (45, 46).

Acupuncture, a traditional Chinese medical procedure that involves inserting small needles into specific body areas, has been studied as a non-pharmacological therapy for pain in a number of clinical settings. Acupressure, or the application of pressure to particular points, is a more easy and accessible alternative to acupuncture (47). A systemic review conducted by Vickers et al. (2012) discovered that acupuncture significantly lowers acute and chronic pain. The benefits of acupuncture in emergency situations are consistent with these results. The analysis emphasized acupuncture’s potential to minimize opioid usage and speed up pain relief, especially for patients suffering from musculoskeletal pain or postoperative pain (48).

Additionally, heat and cold treatment are simple, inexpensive, and well-established nonpharmacological remedies that are commonly used in emergency settings. These methods are very effective in treating musculoskeletal pain, sprains, strains, and other soft tissue injuries that are often seen in the emergency room (48). Heat treatment has been used for centuries and may be provided in a number of methods, including heat lamps, hot baths, and heat pillows. These treatments work at varied intensities to relieve pain, increase blood flow, and decrease muscular tension (49). For example, Freiwald et al. (2021) found that heat treatment effectively relieved pain and muscular stiffness in individuals with non-specific low back pain or muscle strains (50). In contrast, cold therapy is widely used as the first line of treatment for acute injuries because it lowers inflammation and edema, numbs the affected region, and limits pain transmission. Its effectiveness in alleviating acute pain caused by soft tissue injuries in the emergency room has been well proven (49). Further, heat and cold treatments are both non-invasive, inexpensive, and need little training for emergency room physicians, making them ideal for quick pain relief in emergency settings (51).

Transcutaneous Electrical Nerve Stimulation (TENS) is another non-invasive technique gaining favor in emergency settings. TENS alleviates pain perception by activating sensory neurons with electrical impulses (52). Johnson et al. (2015) investigated the utility of TENS for pain treatment in emergency rooms. The research discovered that individuals with acute pain, especially those with soft tissue injuries and musculoskeletal discomfort, had significant pain relief. The theorized mechanism for TENS effectiveness includes the gating of pain signals via non-pain sensory fiber activation and endorphin release (51).

Additionally, virtual reality (VR) effectively diverts patients from their suffering experience by engaging them in immersive digital environments. These approaches have shown promise for pain reduction, notably in pediatric and burn patients. Nonetheless, several research suggest that it may have advantages for adult patients as well (53). Maani et al. (2011) performed a controlled study in adult patients to assess VR’s effectiveness in lowering pain perception during wound care and other treatments. The research found significant decreases in both acute pain and anxiety among individuals undergoing unpleasant procedures (54). Table 1 summarizes the pain management protocols in pediatric and adults in the ER.

**Table 1:** Comparison of pain management strategies in pediatric and adult emergency medicine (created by the authors based on synthesized findings from the reviewed literature).

Line of treatment	Pediatric patients (≤18 years)	Adult patients (>18 years)	Special considerations
First-line analgesics	Acetaminophen; Ibuprofen (weight-based dosing)	Acetaminophen; NSAIDs (e.g., ibuprofen, ketorolac)	Pediatrics: dosing accuracy critical; caution in dehydration and renal disease (34). Adults: NSAIDs contraindicated in renal impairment, peptic ulcer disease, and bleeding risk (37).
Opioids (moderate–severe pain)	Morphine, fentanyl (intranasal or IV; weight-based dosing)	Morphine, fentanyl, hydromorphone	Pediatrics: higher risk of respiratory depression, require close monitoring (35, 55). Adults: risk of dependence, sedation, respiratory depression, particularly in older adult and comorbid patients (39, 56).
Route-specific analgesia	Intranasal fentanyl commonly used for rapid pain relief	IV or oral opioids preferred	Intranasal route reduces need for IV access and procedural distress in children (55). Adults generally tolerate IV/oral routes better (37).
Regional anesthesia/nerve blocks	Increasing use (e.g., femoral nerve block)	Commonly used for trauma and postoperative pain	Contraindicated in coagulopathy or local infection; reduces systemic opioid exposure (36, 38, 56).
Adjuvant pharmacologic agents	Limited use; selected cases (e.g., low-dose ketamine)	Gabapentinoids, antidepressants, corticosteroids	Pediatric use limited due to safety and dosing data (57). Adults tolerate broader adjuvant options for neuropathic or chronic pain (38, 58).
Multimodal analgesia	Combination of acetaminophen, NSAIDs, local anesthetics, non-pharmacological strategies	Combination of NSAIDs, acetaminophen, opioids, and adjuvants	Reduces opioid requirements and improves pain control in both populations (38, 39).
Topical and local analgesia	EMLA cream, local anesthetics for procedures	Local anesthetic infiltration, topical agents	Especially beneficial in pediatrics for needle-related procedures; minimal systemic effects (36, 59).
Non-pharmacological interventions	Distraction, parental presence, play therapy, cold/heat	CBT, relaxation, mindfulness, acupuncture, TENS	Particularly emphasized in pediatrics; effective adjuncts in adults to reduce opioid use (42, 45, 47).
Special populations	Neonates, infants, developmentally delayed children	Elderly, cognitively impaired, comorbid patients	Observational pain scales required; individualized dosing and reassessment essential (33, 36, 60).

ED, emergency department; NSAIDs, non-steroidal anti-inflammatory drugs; IV, intravenous; IN, intranasal; CBT, cognitive behavioral therapy; TENS, transcutaneous electrical nerve stimulation; EMLA, eutectic mixture of local anesthetics; NPRS, Numeric Pain Rating Scale; VR, virtual reality.

## 6 Guidelines and protocols in pediatric versus adult pain management

### 6.1 Pediatric-specific guidelines

Pain management in pediatric emergency care involves prompt and accurate response to minimize discomfort and side effects. Emergency department-specific pediatric pain management guidelines stress timely use of tools of pediatric pain, prompt analgesic therapy, and regular re-assessment of children, as acute presentations of pain are ever changing in nature (26, 59, 60).

Pain severity has been used to guide pain management in the Emergency department, with mild pain often treated with non-pharmacological interventions and non-opioid analgesic agents, which may include acetaminophen or ibuprofen, with dosing based on patient weight. For moderate pain, especially in emergency cases like trauma, burns, and surgical pain, low doses of opiates, especially in cases of delayed establishment of intravenous (IV) access, and intranasal analgesic agents may be used. Severe pain, especially in emergency cases, can only be treated with IV opiates, especially in trauma, burns, and surgical pain, in combination with non-opioid agents (55, 61).

Emergency pediatric pain protocols once again reinforce the importance of minimization of pain during procedures by the administration of topical anesthetizers, the use of distractions, and the role of the caregiver. Monitors for potential side effects, such as respiratory depression, are to be emphasized in the context of the rapid turnover of patients expected in the Emergency department, with the constraints of time posing potential risks for the safety of patients (1, 62).

### 6.2 Adult-specific guidelines

**Figure 2:**
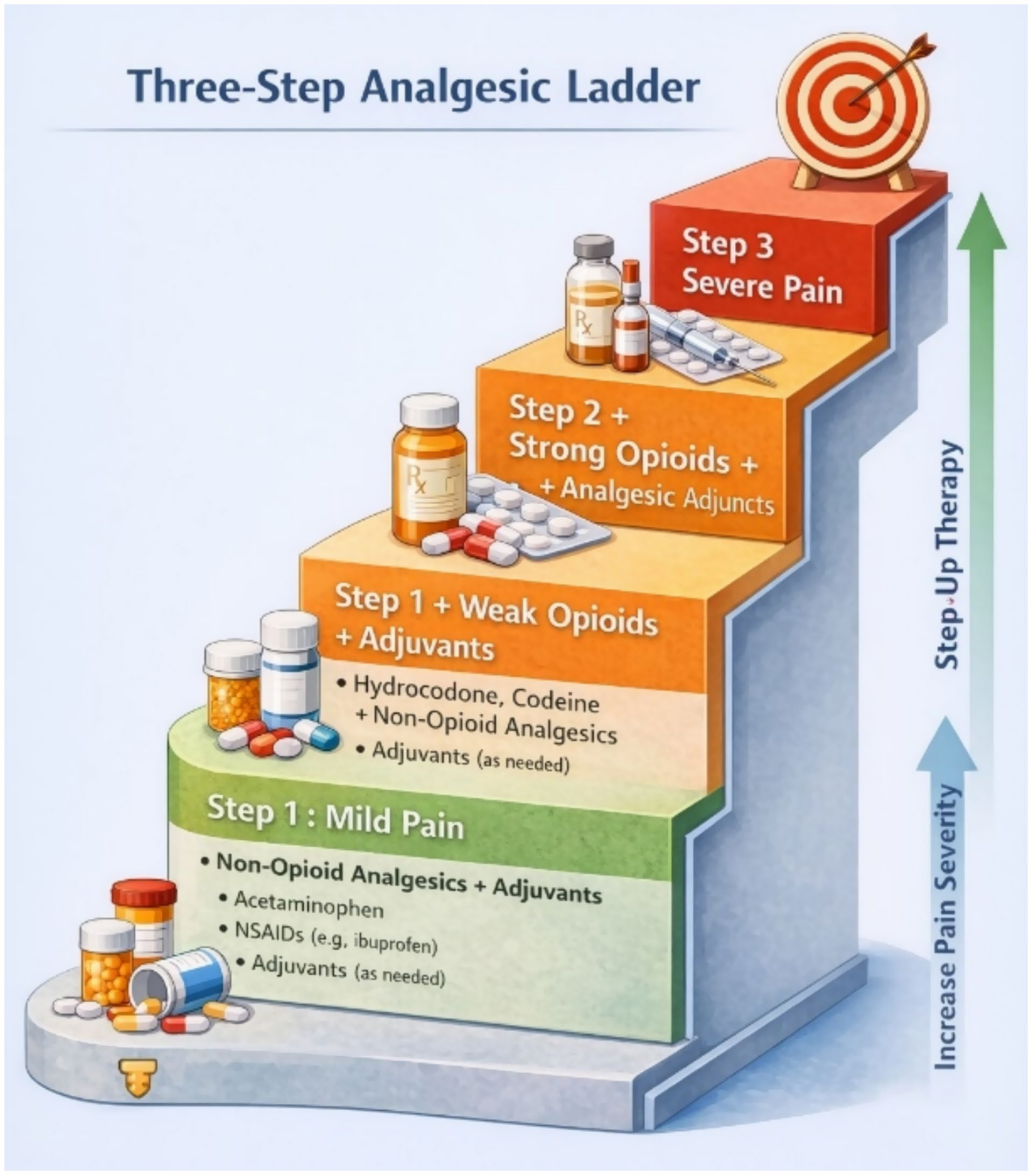
Three-step analgesic ladder (adapted from the World Health Organization analgesic ladder framework).

In 1986, the World Health Organization (WHO) released guidelines for the management of cancer pain, presenting a Three-Step Analgesic Ladder that categorizes pain treatment according to intensity (63) (Figure 2). The ladder classifies analgesic interventions in the following manner: Step 1 entails the administration of non-opioids, potentially augmented with adjuvant analgesics for mild pain; Step 2 advocates for weak opioids in conjunction with non-opioids and adjuvants for mild to moderate pain; and Step 3 prescribes strong opioids paired with non-opioids and adjuvant analgesics for moderate to severe pain. The guidelines urge increasing therapy dosages while pain continues and suggest reducing doses or tapering down when adverse reactions occur. The ladder highlights five fundamental principles: delivering analgesics orally whenever possible, on a scheduled basis, in accordance with the ladder’s hierarchy, customized for the specific patient, and with meticulous attention to detail (63, 64). Although the WHO ladder has been used to manage cancer pain, it is also useful for managing acute and chronic pain requiring analgesics. Nevertheless, the original WHO guidelines significantly downplay the importance of non-pharmacological and non-opioid therapy (64).

The stepwise approach to analgesic therapy described above aligns primarily with the WHO three-step analgesic ladder, which recommends escalation from non-opioid analgesics to weak opioids and subsequently strong opioids depending on pain severity. In contrast, the Centers for Disease Control and Prevention (CDC) 2016 and updated 2022 guidelines focus primarily on opioid prescribing practices, emphasizing the prioritization of non-opioid therapies and careful risk–benefit assessment when opioids are considered.

The 2016 Centers for Disease Control and Prevention (CDC) guideline for prescribing opioids for chronic pain was designed for primary care clinicians, such as physicians, nurse practitioners, and physician assistants. It specifically addresses the management of chronic pain, defined as pain lasting longer than 3 months or past the expected duration of tissue healing. The guideline is designed to be applicable to patients aged 18 and older who are experiencing chronic pain in the absence of active cancer treatment, palliative care, or end-of-life care. The recommendations may be applicable to acute care settings or other specialists, such as emergency physicians (58).

The stepwise approach to analgesic therapy described above aligns with the WHO three-step analgesic ladder, which recommends escalation from non-opioid analgesics to weak opioids and subsequently strong opioids based on pain severity. In contrast, the CDC 2016 guideline, updated in 2022, does not follow a stepwise model but instead emphasizes the prioritization of non-pharmacological and non-opioid therapies for chronic non-cancer pain. According to these guidelines, opioid therapy should only be considered when the expected benefits outweigh the potential risks. When opioids are prescribed, they should be initiated at the lowest effective dose and for the shortest appropriate duration, particularly in acute pain settings. The guidelines also recommend careful monitoring, reassessment within 1 to 3 months of initiation, and tapering when the risks of continued opioid use outweigh the benefits. This approach supports individualized, patient-centered decision-making in pain management (56, 58, 65).

## 7 Challenges in implementing pain management protocols

Pain management in the Emergency Department is uniquely challenging, given the acute, high-pressure model of care delivery, overcrowding, lack of time to conduct a detailed evaluation, and the urgent need to attend to life-endangering disorders, etc. In contrast to the inpatient model, ER pain management clinicians are often obligated to determine pain pharmacologic management with limited information, with the concurrent necessity to address a multitude of patients with diverse pain disorders, thereby mandating a disconnection between guidelines and practice, which remains an ongoing concern to the present time (57, 62).

### 7.1 Pediatric-specific challenges

Pediatric pain management in the Emergency Department is particularly complex, as children commonly present with acute injuries or illnesses while experiencing fear, anxiety, and unfamiliar surroundings. Pain in pediatric emergency patients is frequently underestimated and undertreated, partly due to difficulties in rapid assessment and limited verbal communication, especially in infants and young children (62). In busy emergency environments, validated pediatric pain scales may be underutilized, leading to reliance on subjective clinical judgment rather than standardized assessment tools tailored to developmental stage (57).

Time constraints, the requirement for rapid triage, and barriers to communication frequently impede optimal assessment of pain intensity and location, and proxy measures via behavioral observation scales are often relied on in preverbal children. Physicians working in emergency departments may have less access to specific pediatric pharmacologic advice about opioids, and there is a general lack of practical guidance relating to dosing regimens, adverse effects profile, and indications, which contributes to concern or inconsistency about analgesic prescribing. The episodic nature of Emergency Department encounters further limits opportunities for follow-up and reassessment, important components of effective pediatric pain management (57, 66).

Also, parental anxiety levels, along with misapprehensions about analgesics, especially opioids, can prolong the giving of consent for such procedures or lead to denial of appropriate analgesics, even if required. The fastidious environment of an Emergency Department can leave little room to tackle the anxiety of parents, thereby creating an issue in the management of children’s analgesia needs, thereby causing issues in managing children’s analgesia needs well (62, 66, 67). Lack of proper analgesia in pediatric Emergency Department can result, apart from acute problems, in chronic problems such as fear of future medical encounters, chronic pain, and avoidance of future medical interventions (60, 66).

### 7.2 Adult-specific challenges

These same operational pressures, diagnostic uncertainty, and heterogeneity of the patient population serve to impede adult pain management in the Emergency Department. Emergency presentations are often characterized by complex etiologies, comorbidities, and polypharmacy, raising specific challenges with respect to rapid and individualized analgesic decision-making. Limited familiarity with contemporary pain protocols among clinicians, high patient volumes and bed crowding, contribute to inconsistent application of standardized pain assessment tools and delays in the initiation of appropriate therapy (68, 69).

Similarly, there are challenges in the management of patients with impaired cognition, mental health problems, and nervous conditions in which pain is atypical and not dependable. In such conditions, the healthcare professionals in the Emergency Department need more sophisticated attention, but since pain is acute in nature, the lack of sufficient observation in the Emergency Department creates a major problem (70–72). Moreover, issues concerning opioid abuse add to the problems in pain management.

In addition to patient-related factors, the level of knowledge regarding pain management strategies available to the patient, inadequate patient involvement in the decision-making process, and the lack of communication during the short visit in the Emergency Department also have an impact on patient satisfaction and adherence to pain management strategies (68). In addition to those aspects identified in earlier studies, cultural issues, the language barrier, and economic issues have also an impact on the willingness of the patient to comply with the pain management strategies (72). Fragmented continuity of care in the case of recurrent pain patients seeking pain management in the ER, in addition to poor adherence to outpatient pain management strategies, adversely affects the pain management outcomes (73).

Overall, the fast-paced environment of the ER, combined with provider, patient, and system-level barriers, still hampers consistent implementation of evidence-based pain management protocols among both pediatric and adult populations. Large-scale ED-specific training programs need to be taken on board, along with streamlining assessment pathways, increasing the availability of assessment tools, and embedding multidisciplinary approaches into the flow of patient care.

## 8 Comparison of outcomes

### 8.1 Psychological and emotional impact

Pain is a complicated sensory and emotional experience that varies between individuals, and even within individuals, depending on the context and significance of the pain, as well as the person’s psychological state. Clinical and experimental studies show that even simple psychological interventions, like distraction, can significantly alter pain perception. The impact of pain management on mental health differs between children and adults, with far-reaching consequences for both groups (74–76).

For children, effective pain management is crucial for healthy psychological development and promotes long-term positive mental health outcomes. In contrast, pain management for adults often focuses on maintaining the ability to work and fulfill responsibilities, and preventing disability or loss of independence (76). A study published in *The Lancet Regional Health - Europe* underscores the potential risks associated with long-term painkiller usage in childhood. Individuals under the age of 25 who experience pain are 29% more likely to have a mental disorder later in life. Furthermore, children treated with prescription pain medications are 46% more likely to have a mental disease in adulthood, and an 82% higher risk of substance abuse (77). Another study showed the relationship between pain management and mental health varies across age groups. Pain intensity, effect on mental health is more pronounced in individuals 59 years or younger, but not in those 60 or older. These finding were consistent with theories of socioemotional selectivity and affect regulation, which suggest that older adults engage in more effective regulation of emotion; they prioritize meaningful experiences and do so by shifting their social relationships to those that are more positive, and they alter their attention, beliefs and environments so as to maximize positive and dampen negative affect. As a result, older adults report lower depression and anxiety than those who are younger. Thus, the ability to process and express emotions may not be particularly relevant or helpful to older adults. In contrast, these abilities may be particularly helpful for adults of age 59 or less with pain, who tend to have greater negative affectivity and need for improved emotion regulation (78).

From a developmental perspective, pain in childhood can have lasting effects on adult health and well-being. Multiple studies have demonstrated that childhood pain predicts depression and anxiety disorders in adulthood, even after controlling for co-occurring psychopathology in childhood. For example, in a population-based community sample, the presence of adolescent chronic pain was associated with higher subsequently reported rates of lifetime anxiety disorders (21.1% vs. 12.4%) and depressive disorders (24.5% vs. 14.1%) in adulthood compared with individuals without a history of adolescent chronic pain (76). Any emergency medical system that provides treatment for children should have a demonstrated quality improvement program in which review of sedation and pain management practices in pediatric patients takes place at regular intervals (79).

### 8.2 Quality of life and recovery

The effective management of pain is a cornerstone factor affecting the quality of life and recovery outcomes, not only among adult but also among pediatric patients presenting to the Emergency Department. Pain is a deleterious factor affecting individuals across various ages; it does have different long-term and short-term outcomes on pediatric and adult patients.

Thus, it is clear that reduced analgesia can significantly impair the development of pediatric emergency patients, leading to important psychological consequences, such as increases in future pain sensitivity, anxiety about medical procedures, and avoidance behaviors that can be seen well into adulthood. Acute pain management in children has been related to future negative mental health problems, including anxiety, depression, and mood-related illnesses that occur during adulthood, such that early childhood analgesia has been connected to a significantly increased risk of future anxiety, depression, mood, substance, and psychotic disorders, thereby emphasizing the need for safe use of prescription analgesics in pediatric emergency care that actually promotes a high quality of life in children beyond the need to relieve acute discomfort in the pediatric emergency department (76, 77).

In adult patients presenting to the emergency department, effective pain management plays a critical role in maintaining functional capacity, independence, and preventing disability. Uncontrolled acute pain can negatively impact mobilization, sleep, and rehabilitation, as well as social and occupational functioning. Several studies have demonstrated that pain is associated with reduced physical activity, decreased work capacity, and poorer quality of life. Conversely, timely and appropriate pain management in emergency settings has been shown to improve ambulation, functional recovery, and overall emotional well-being, thereby contributing to improved patient outcomes (80–83).

Lastly, differences with regard to the emotional response to pain, the healing process after getting emergency medical treatment for pain, would likely be seen between younger adults and older adults. For instance, younger adults may be expected to show the tendency to associate pain with negative psychological states, while older adults may show the tendency to be emotionally resilient despite the pain, which could help to minimize the effects of pain with regard to quality of life (78, 79). Table 2 presents representative studies included in this narrative review, highlighting key findings related to pain assessment, analgesic strategies, and pain management protocols in pediatric and adult emergency medicine. These studies were selected to illustrate the major themes identified during the synthesis rather than to provide an exhaustive list of all included studies.

**Table 2:** Representative studies illustrating key findings in pediatric and adult pain management in emergency medicine (created by the authors based on synthesized findings from the reviewed literature).

Author	Study focus	Population	Key findings
Yam et al. (2018) (4)	Pain pathways physiology	General population	Identified major nociceptive pathways and neurotransmitters involved in pain signaling
El Tumi et al. (2017) (11)	Age-related pain sensitivity	Pediatric vs. adult	Pain thresholds increase with age due to maturation of neural pathways
Gai et al. (2020)(26)	Pediatric pain assessment	Pediatric patients	Validated use of FLACC and other behavioral scales
Kang and Demiris (2018) (30)	Adult pain assessment tools	Adult population	VAS and NPRS commonly used for self-reported pain intensity
Murphy et al. (2014) (55)	Intranasal fentanyl	Pediatric emergency patients	Demonstrated rapid analgesic effect and improved procedural tolerance
Dowell et al. (2022) (56)	Opioid prescribing guidelines	Adult patients	Recommended prioritization of non-opioid therapies

FLACC, face, legs, activity, cry, consolability scale; VAS, visual analog scale; NPRS, numeric pain rating scale.

## 9 Strengths and limitations

One of best attributes of this narrative review lies in its ability to give a detailed comparison of managing pain in children and adults in an emergency setting, incorporating physiological, as well as psychosocial, factors in understanding it better. However, it can also be limited in certain sections, such as incorporating ‘heterogeneous’ data, lack of quantitative analysis, as well as differences in designs of various studies.

## 10 Conclusion

This comparative analysis of the pain management protocols in emergency medicine for pediatric and adult populations points to the challenge of addressing pain in such diverse age groups. Significant differences in physiological pain perception, assessment tools, and treatment strategies justify emphasizing tailored approaches reflecting particular needs in each demographic group. Pediatric protocols must take into account developmental stages, communication barriers, and heightened sensitivity to medication, whereas adult protocols must navigate issues related to comorbidities and the risk of opioid dependency. Despite the many advances that have been achieved in pain management, significant challenges persist in accurately assessing pain in patients who are unable to communicate effectively and in applying evidence-based guidelines consistently in high-intensity emergency settings.

## 11 Recommendations and prospectives

Emergency departments should adopt standardized, age-appropriate pain assessment tools and implement multimodal, patient-centered analgesic pathways tailored to pediatric and adult populations. Ongoing staff education, early pain assessment at triage, regular reassessment, and greater integration of non-pharmacological interventions are essential. Future research should focus on Emergency Department-specific outcomes, protocol implementation strategies, and long-term effects of early pain management across age groups to optimize quality of care in emergency medicine.

Although the pain management principles discussed in this review are universally applicable in the context of emergency medicine, their application may vary depending on the regional infrastructure and resource availability. In the context of developed healthcare systems, more advanced multimodal pain management strategies, regional anesthesia, and technology-based strategies such as virtual reality may be more feasible. In the context of low- and middle-income countries, emergency departments may be more reliant on cost-effective pharmacologic strategies and simple non-pharmacologic strategies such as distraction, reassurance, and cold or heat therapy.

Therefore, the findings of this review must be interpreted in the context of both global and regional realities, and attempts should be made to apply pain management strategies based on regional resource availability.
